# The complete mitochondrial genome of an egg parasitoid (*Trichogramma chilonis*)

**DOI:** 10.1080/23802359.2021.1947915

**Published:** 2021-07-12

**Authors:** Yanlong Liu, Baoqian Lyu, Xue Tang, Hui Lu, Jihong Tang, Rui Meng, Bo Cai

**Affiliations:** a China Academy of Tropical Agriculture Sciences/Key Laboratory of Integrated Pest Management on Tropical Crops, Ministry of Agriculture and Rural Affairs, Environment and Plant Protection Institute, Haikou, China; b College of Agriculture, Heilongjiang Bayi Agriculture University, Heilongjiang, China; c Post-Entry Quarantine Station for Tropical Plant, Haikou Customs District P. R. China/Hainan Province Engineering Research Center for Quarantine, Prevention and Control of Exotic Pests, Haikou, P. R. China

**Keywords:** *Trichogramma chilonis*, Chalcidoidea, mitochondrial genome, phylogenetic relationship

## Abstract

*Trichogramma chilonis* Ishii is an important natural enemy of several lepidopterous pests on crops. In this study, we sequenced the complete mitochondrial genome of *T. chilonis* (GenBank accession number MW789210). The length of the complete mitochondrial genome was 16,147 bp, containing 13 protein-coding genes, 22 tRNA genes, 2 rRNA genes, and a non-coding control region. The overall base composition of the genome in descending order was 44.8% T, 41.8% A, 9.0% G and 4.5% C, with a significant AT bias of 86.6%. Phylogenetic analysis indicated that *T. chilonis* had a close relationship with *Trichogramma ostriniae*.

*Trichogramma chilonis* (Ishii 1941) (Trichogrammatidae: Chalcidoidea) is an important natural enemy of several lepidopterous pests that feed on crops and vegetables (Nadeem et al. 2010). It plays an important role in integrated pest management around the world (Smith 1996; Ahmad et al. 1998). *T. chilonis* is an endoparasitic insect with strong reproductive ability (Kerima et al. 2018). It can attack the eggs of more than 200 species of insects. Therefore, the large-scale breeding and release of *T. chilonis* is an economically viable method for the control of a variety of lepidopteran pests (Edwin et al. 2016). In China, *T*. *chilonis* is widely distributed and is employed in integrated pest management for maize, rice, sugarcane and other crops (Yi et al. 2014). There are many studies on *T*. *chilonis*, including mass rearing, increase of parasitism rate and chemoreception (Fatima et al. 2002; Liu et al. 2016); however, little research has been done on the genome of *T*. *chilonis*. In order to provide some biological data for *T*. *chilonis*, it is necessary to carry out some germplasm analysis. Here, we firstly reported the complete mitochondrial genome of *T*. *chilonis*, which will provide useful information for further studies on population genetics, phylogenetic construction and other relevant studies in *T*. *chilonis*.

In this study, a specimen of *T*. *chilonis* was collected from Danzhou, Hainan province and reared in the Environment and Plant Protection Institute, China Academy of Tropical Agriculture Sciences, Hainan, China (110°20′9″N, 19°59′21″E). Voucher specimens (K30-S03-R118) were preserved in 95% ethanol and deposited at herbarium of Post-Entry Quarantine Station for Tropical Plant, Haikou Customs District P.R. China (URL, Meng Rui, huamei0391@163.com). The total genomic DNA was extracted by a Genomic DNA Extraction Kit (Tiangen Biotech, Beijing, China), following the manufacturer’s instruction. The assembly method used here adopts the method of Meng et al. (2019), and uses the Mitoz software package to conduct the assembly with default parameters. The mitogenome was sequenced using the Illumina HiSeq X TEN Sequencing System 2500 platform with 150 bp paired-end reads. The annotations were mainly compared with the existing mitochondrial genomes of related species, and the annotation results were confirmed and modified by MITOS online tool (Bernt et al. 2013).

The total length of the mitochondrial genome sequence of *T*. *chilonis* was 16,147 bp, with the base composition of 41.8% A, 44.8% T, 4.5% C, and 9.0% G. It is highly A + T biased, accounting for 86.6%, showing strong AT skew. It comprised 2 ribosomal RNA genes, 13 protein-coding genes, 22 transfer RNA genes and one AT-rich region with a length of 668 bp. All tRNA genes can fold into a typical cloverleaf structure, with lengths ranging from 63 to 70 bp. The 12S rRNA (777 bp) and 16S rRNA genes (1391 bp) are located between *trnV* and *trnG* and between *trnA* and *trnL1*, respectively. All protein-coding genes were initiated with ATN (ATA/ATG/ATT). Ten coding genes use TAN as the termination codons (nd1, nd4, nd5, cox2, atp8, atp6, cox3, cox1 and nd2 with TAA; nd4l with TAG), whereas nd3 end with ATA, cytb end with CAT and nd6 end with GAT.

Phylogenetic analysis of 13 mitochondrial protein-coding genes of *T*. *chilonis* and other 18 species in Chalcidoidea was performed by Bayesian inference and maximum likelihood in Physosuite (Nguyen et al. 2015; Zhang et al. 2020). The phylogenetic analysis was constructed with 20 different species of lepidopterous using neighbor-joining tree model with 5000 bootstrap replicates, and these genes are concatenated. The result showed that *T*. *chilonis* belongs to Trichogrammatidae (Figure 1). We expect that the present result can contribute to molecular identification of this species and be helpful to explore the phylogeny of Chalcidoidea.

**Figure 1.**
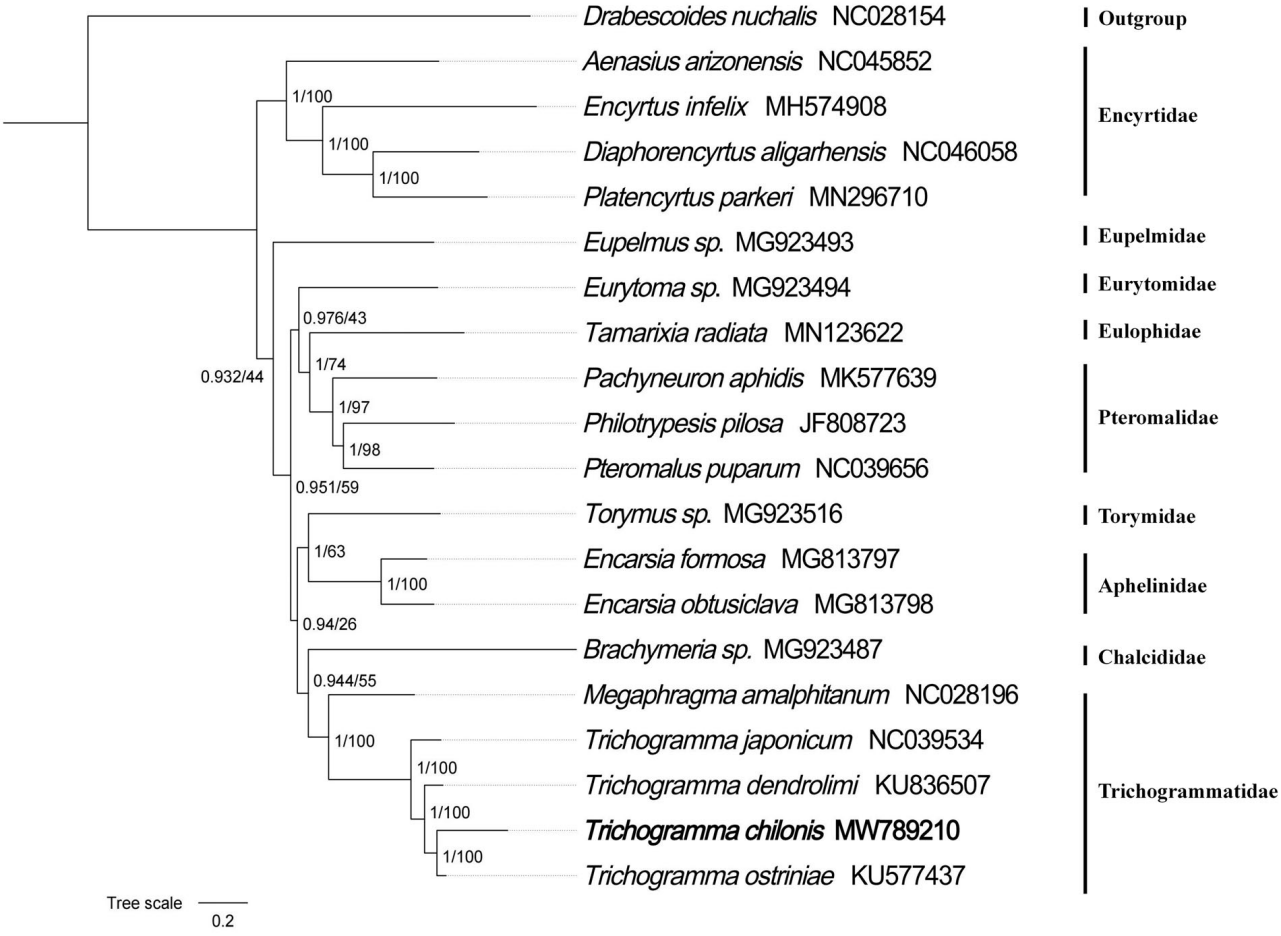
Phylogenetic relationships of 13 mitochondrial protein-coding genes within Chalcidoidea was performed using Bayesian/ML methods. Mitochondrial phylogeny of *T. chilonis* and other Trichogrammatidae spedes. Numbers on branches are Bayesian posterior probabilities (left) and bootstrap values (right).

## Data Availability

The genome sequence data that support the findings of this study are openly available in GenBank of NCBI at (https://www.ncbi.nlm.nih.gov/) under the accession no. MW 789210.The associated BioProject, SRA, and Bio-Sample numbers are PRJNA719932, SRR14149001, and SAMN18628701 respectively.

## References

[CIT0001] Ahmad N, Ashraf M, Fatima B., Nasrullah 1998. Potential of *Trichogramma chilonis* to parasitize eggs of pink, spotted and spiny bollworms of cotton. Pak J Zool. 30(1):39–40.

[CIT0002] Bernt M, Donath A, Jühling F, Externbrink F, Florentz C, Fritzsch G, Pütz J, Middendorf M, Stadler PF. 2013. MITOS: improved de novo metazoan mitochondrial genome annotation. Mol Phylogenet Evol. 69(2):313–319. 22982435 10.1016/j.ympev.2012.08.023

[CIT0003] Edwin ES, Vasantha-Srinivasan P, Ponsankar A, Thanigaivel A, Selin-Rani SW, Mankin R, Senthil-Nathan S, Kalaivani K, Kalaivani RK, Duraipandiyan V, et al. 2016. Effects of temperature and nonionizing ultraviolet radiation treatments of eggs of five host insects on production of *Trichogramma chilonis* Ishii (Hymenoptera: Trichogrammatidae) for biological control applications. J Asia-Pac Entomol. 19(4):1139–1144.

[CIT0004] Fatima B, Ashraf M, Ahmad N, Suleman N. 2002. Mass production of Trichogramma chilonis, an economical and advanced technique. Paper presented at: BCPC Conference: Pests & Diseases.

[CIT0005] Kerima OZ, Niranjana P, Vinay Kumar BS, Ramachandrappa R, Puttappa S, Lalitha Y, Jalali SK, Ballal CR, Thulasiram HV. 2018. De novo transcriptome analysis of the egg parasitoid *Trichogramma chilonis* ishii (hymenoptera: trichogrammatidae): a biological control agent. Gene Rep. 13:115–129.

[CIT0006] Liu B, Yang L, Yang F, Wang Q, Yang Y, Lu Y, Gardiner MM. 2016. Landscape diversity enhances parasitism of cotton bollworm (*Helicoverpa armigera*) eggs by *Trichogramma chilonis* in cotton. Biol Control. 93:15–23.

[CIT0007] Meng R, He Y, Ao S, Cai B. 2019. Complete mitochondrial genome of the coconut black-headed caterpillar Opisina arenosella (Lepidoptera: Gelechioidea: Xyloryctidae). Mitochondrial DNA Part B. 4(1):1237–1238.

[CIT0008] Nadeem S, Ashfaq M, Hamed M, Ahmed S. 2010. Optimization of short and long term storage duration for *Trichogramma chilonis* (Ishii) (Hymenoptera: Trichogrammatidae) at low temperatures. Pak J Zool. 42(1):63–67.

[CIT0009] Nguyen LT, Schmidt HA, Haeseler VA, Minh BQ. 2015. IQ-TREE: a fast and effective stochastic algorithm for estimating maximum-likelihood phylogenies. Mol Biol Evol. 32(1):268–274. 25371430 10.1093/molbev/msu300 PMC4271533

[CIT0010] Smith SM. 1996. Biological control with *Trichogramma*: advances, successes, and potential of their use. Annu Rev Entomol. 41 (1):375–406. 15012334 10.1146/annurev.en.41.010196.002111

[CIT0011] Yi DW, Xiao R, Zhao YL, Song LD, Zhang GR. 2014. Cold storage of *Corcyra cephalonica* eggs affects the quality of *Trichogramma chilonis* offspring. J Environ Entomol. 36(4):565–571.

[CIT0012] Zhang D, Gao FL, Li WX, Jakovlić I, Zou H, Zhang J, Wang GT. 2020. PhyloSuite: an integrated and scalable desktop platform for streamlined molecular sequence data management and evolutionary phylogenetics studies. Mol Ecol Resour. 20(1):348–355. 31599058 10.1111/1755-0998.13096

